# How to Choose Platelet-Rich Plasma or Hyaluronic Acid for the Treatment of Knee Osteoarthritis in Overweight or Obese Patients: A Meta-Analysis

**DOI:** 10.1155/2020/7587936

**Published:** 2020-03-10

**Authors:** Pan Luo, Zhencheng Xiong, Wei Sun, Lijun Shi, Fuqiang Gao, Zirong Li

**Affiliations:** ^1^Peking Union Medical College, Chinese Academy of Medical Sciences, Graduate School of Peking Union Medical College, Department of Orthopedics, China-Japan Friendship Hospital, China-Japan Friendship Institute of Clinical Medicine, Beijing 100029, China; ^2^Department of Orthopedics, China-Japan Friendship Hospital, National Health and Family Planning Commission of the People's Republic of China, Beijing 100029, China

## Abstract

**Objective:**

The purpose of this meta-analysis was to determine whether platelet-rich plasma (PRP) was better than hyaluronic acid (HA) for the treatment of knee osteoarthritis (OA) in overweight or obese patients.

**Design:**

Two reviewers independently used the keywords combined with free words to search English-based electronic databases according to Cochrane Collaboration guidelines, such as PubMed, Embase, ScienceDirect, and Cochrane library. The pooled data were analyzed using RevMan 5.3.

**Results:**

Ten randomized controlled trials (RCTs) with 1096 patients were included. During the first two months of follow-up, there was no significant difference between the two groups. At the 3rd, 6th, and 12th months of follow-up, the pooled analysis showed that PRP was better than HA for the treatment of knee OA in overweight or obese patients. There were significant differences between the two groups at Western Ontario and McMaster Universities Osteoarthritis Index (WOMAC) total score (3 months: MD = −1.35, [95% CI: −2.19 to −0.50], *P*=0.002, *I*^2^ = 0%; 6 months: MD = −7.62, [95% CI: −13.51 to −1.72], *P*=0.01, *I*^2^ = 88%; 12 months: MD = −12.11, [95% CI: −20.21 to −4.01], *P*=0.003, *I*^2^ = 94%).

**Conclusions:**

For overweight or obese patients with knee OA, intra-articular injection of PRP in a short time was not necessarily superior to HA, but long-term use was better than HA in pain and functional relief.

## 1. Introduction

Knee OA is a chronic disease caused by a variety of causes, characterized by degeneration of articular cartilage, which has an adverse impact on the quality of life of patients [1]. Moreover, articular cartilage regeneration is very difficult; once damaged, it is difficult to repair [2]. It is also one of the most common causes of pain and disability in adults. This disease mostly occurs in the elderly, more women than men, 65 years old patients with knee OA prevalence of 50% [3]. Obesity is an important factor in the development of OA [4]. According to the recommendation of WHO, the patients with BMI ≥25 Kg/m^2^ were defined as overweight, and the patients with BMI ≥30 Kg/m^2^ were defined as obese [4].

There are many conservative treatments for OA, such as drug therapy, intra-articular injection of HA and PRP [5], physical therapy [6], and ozone therapy [7]. All kinds of treatments aim at relieving knee pain and improving joint mobility [8]. Intra-articular HA injection is widely used in the treatment of knee OA. Its viscosity-inducing properties increase joint lubrication and provide therapeutic effects, which have been reported in many studies and meta-analysis [9]. In addition, PRP, as biologic therapy, has become an interesting treatment option to improve the joint status of patients with OA [10].

Many studies had compared the effects of PRP and HA in patients with knee OA [5, 11–19]. For example, in one study, there was no difference between HA and PRP at any time point on pain relief in patients with knee OA [5]. In one study, PRP was shown to be superior to HA in the short-term relief of early OA symptoms of the knee [11]. Moreover, no meta-analysis has been conducted on the efficacy of HA and PRP in overweight or obese patients with knee OA. Therefore, our goal was to compare the efficacy of the two treatment methods in patients with knee OA with body mass index (BMI) ≥25 Kg/m^2^ by meta-analysis.

## 2. Methods

We carried out this meta-analysis in accordance with the Preferred Reporting Items for Systematic Reviews and Meta-Analyses (PRISMA) [20].

### 2.1. Search Strategy

In order to obtain all the literature related to our research, in the first step, two reviewers independently used the keywords combined with free words to search English-based electronic databases according to Cochrane Collaboration guidelines, such as PubMed (1966 to December 1, 2019), Embase (1980 to December 1, 2019), ScienceDirect (1980 to December 1, 2019), and Cochrane library (1966 to December 1, 2019). In the second step, the potentially related literature was searched from the list of references of all included studies. We used Medical Subject Headings (MeSH) terms and corresponding keywords to search the following terms “platelet-rich plasma or PRP,” “hyaluronic acid or HA,” “knee osteoarthritis,” “overweight,” and “obesity” with the Boolean operators “AND or OR.” Two researchers independently conducted preliminary screening by reading the titles and abstracts of the retrieved literature. Then, the selected literature should be further filtered by reading the full text as much as possible. All disagreeable literature was resolved after discussion.

### 2.2. Selection Criteria

All trials included in our study meet the following criteria: (1) All studies were original RCTs; (2) the mean BMI of patients for each study was ≥25 Kg/m^2^ [21]; (3) patients were diagnosed with knee OA according to the criteria of American College of Rheumatology with radiographic confirmation (Kellgren-Lawrence score of I-IV or Ahlbäck grades 1 to 3) in all studies [22, 23]; (4) all studies included PRP and HA groups, all of which were intra-articular injections, with a comparison of outcomes between the two groups; (5) the full text of the included literature can be obtained, and the measurement data of WOMAC, International Knee Documentation Committee (IKDC) Subjective Score, Visual Analogue Scale (VAS), and EuroQol visual analogue scale (EQ-VAS) can be extracted [24–26].

The following studies were excluded from the meta-analysis: nonrandomized studies; the patients with BMI <25 kg/m^2^; studies not suitable with the inclusive criteria; and articles for which we were unable to obtain the full text and relevant data for pooled analysis.

### 2.3. Data Extraction

Data were extracted independently by two researchers. After discussion, disagreements in the data extraction process were resolved, and then another researcher used the spreadsheet to collect the data. We extracted the following data: first author, publication year, country, study type, number of participants (PRP : HA), BMI, age, gender, radiographic classification of OA, intervention (PRP : HA), application method, follow-up duration, parameters for evaluation, and outcomes data. A small number of studies did not provide complete data, and we tried to get the original data by contacting the author teams.

### 2.4. Risk of Bias Assessment

The risk of bias in each included RCT was assessed according to the Cochrane Handbook for Systematic Reviews [27]. The evaluation of bias can be divided into 7 sections: random sequence generation, allocation concealment, blinding of participant and personnel, blinding of outcome assessment, incomplete outcome data, selective reporting, and other bias. Each section can have a high risk of bias, low risk of bias, and unclear risk of bias depending on the actual content of the included study [27].

### 2.5. Statistical Analysis

Different studies compared PRP and HA groups according to different follow-up months and outcome measures. We pooled and calculated data of different outcome measures for all studies under the same follow-up month and placed them on the same form. The same outcome measure was divided into subgroups according to the follow-up month. We analyzed continuous data using weighted mean differences (WMD) and their 95% confidence interval (CI), such as WOMAC total score, WOMAC pain score, WOMAC stiffness score, WOMAC physical function score, IKDC, VAS, and EQ-VAS. Statistical heterogeneity was calculated by using a chi-square test and *I*^2^ test. It is considered that the *I*^2^ values of 25%, 50%, and 75% indicate low, moderate, and high heterogeneity, respectively [28]. When *I*^2^ ≤ 50%, we performed a fixed-effect model for the meta-analysis. Otherwise, the random-effect model was performed. Publication bias was assessed by using the funnel plot. The meta-analysis was performed using RevMan 5.3 for Windows (Cochrane Collaboration, Oxford, UK). If the result of the meta-analysis was a probability of *P* < 0.05, it was considered to be statistically significant.

## 3. Results

### 3.1. Literature Search

**Figure 1:**
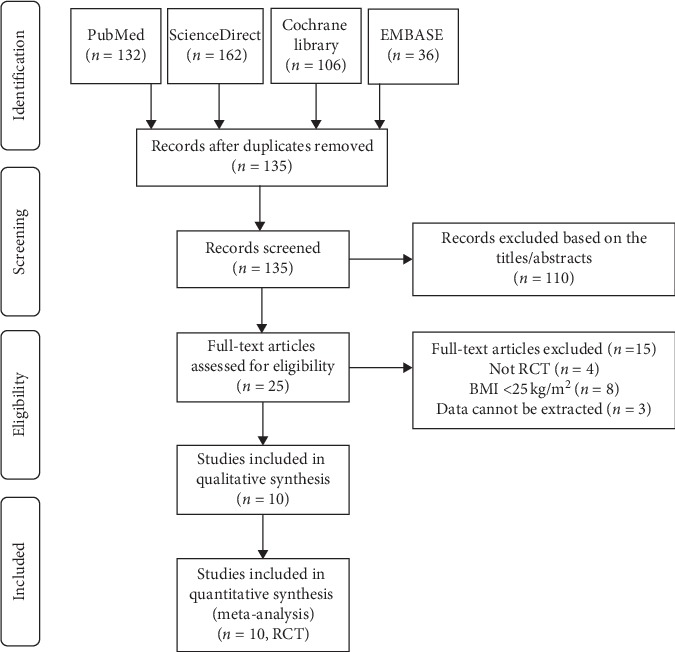
Flow diagram of the study selection process for the meta-analysis.

In the first step, we searched multiple databases and identified 436 records. After removing the duplicate records and the irrelevant records by reading the titles and abstracts, a total of 25 records were selected and the next step was to read the full text. According to the inclusion criteria, records of non-RCT, records with an average BMI <25 Kg/m^2^, and records for which data could not be extracted were excluded. In the end, 10 RCTs were successfully included. The following flow chart showed the search strategy and the process of the study selection (Figure 1) [5, 11–19].

### 3.2. Study Characteristics

This meta-analysis included a total of 10 RCTs published between 2012 and 2018. Characteristics of all the studies included in the meta-analysis are shown in Table 1. All studies compared differences in the therapeutic effects of PRP and HA in overweight or obese patients with knee OA and were followed for a minimum of 1 month to a maximum of 24 months. In these studies, patients in the PRP group were more than patients in the HA group and more female patients than male patients. A total of 9 studies had an average BMI of ≥25 Kg/m^2^ and <30 Kg/m^2^ (overweight level) [5, 11, 13–19], and the remaining study had an average BMI of ≥30 Kg/m^2^ (obesity level) [12]. In nine studies, the severity of OA was classified according to the Kellgren and Lawrence grading scale, and the remaining study was classified according to the Ahlbäck grading scale. Of the 10 studies, PRP for 2 studies was PRGF-Endoret [11, 12], 3 studies used low-molecular-weight HA [5, 14, 19], and 4 studies used high-molecular-weight HA [12, 13, 16, 18].

### 3.3. Risk of Bias

**Figure 2:**
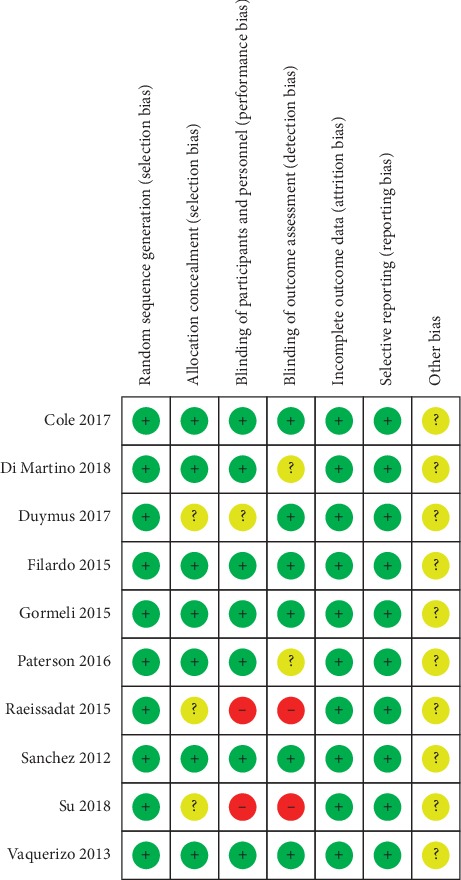
Risk of bias summary: +, low risk of bias; −, high risk of bias; ?, bias unclear.

Of the 10 studies, 8 studies [5, 11–13, 15–18] were considered to have a low risk of bias, while 2 studies [14, 19] were found to have a high risk of bias. Random sequence generation was found in 10 studies. Allocation concealment and blinding of participants and personnel were found in 7 studies [5, 11–13, 15, 17, 18]. Blinding of outcome assessment was found in 6 studies [5, 11–13, 15, 17]. As shown in Figure 2, incomplete outcome data and selective reports were not found in 10 studies.

### 3.4. Comparative Analysis of Therapeutic Effects of PRP and HA

After carefully reading and analyzing the included articles, we summarized the evaluation tools used to measure the effect of patients after receiving PRP or HA, including WOMAC scores, IKDC subjective score, VAS, and EQ-VAS. As shown in Table 2, there are differences in the therapeutic effects of PRP and HA depending on the months of follow-up. This article used WOMAC scores as the primary outcome measurement. Secondary outcome measures were IKDC subjective score, VAS, and EQ-VAS. As the primary outcome measurement, WOMAC scores are composed of three parts: pain, stiffness, and physical function. Therefore, we divide WOMAC in different months into 3 subgroups. At the same time, we performed a subgroup analysis of IKDC, VAS, and WOMAC total score at different time points.

#### 3.4.1. Two Months after Follow-Up

In the first month, a total of 3 studies [15, 16, 19] (143 patients) provided data on VAS for the PRP and HA groups, and a total of 3 studies [5, 16, 19] (221 patients) provided data on WOMAC pain score. There was no significant difference between the two groups according to the results of the pooled analysis (VAS: *P*=0.89, *I*^2^ = 0%; WOMAC pain score: *P*=0.96, *I*^2^ = 16%). A total of 2 studies (122 patients) provided data on WOMAC total score, stiffness score, and physical function score for the PRP and HA groups [16, 19]. Based on the results of the pooled analysis, there was no significant difference between the two groups at WOMAC scores (total score: *P*=0.24, *I*^2^ = 78%; stiffness score: *P*=0.35, *I*^2^ = 35%; physical function score: *P*=0.12, *I*^2^ = 59%) (Table 2). *I*^2^ > 50% in WOMAC total score and physical function score represent high heterogeneity [28]. Heterogeneity may be related to too few inclusion studies, and more research is needed in the future to analyze sources of heterogeneity.

In the second month, a total of 2 studies (350 patients) provided data on EQ-VAS and IKDC score for the PRP and HA groups [13, 18]. There was no significant difference between the two groups according to the results of the pooled analysis (EQ-VAS: *P*=0.12, *I*^2^ = 0%; IKDC: *P*=0.73, *I*^2^ = 0%).

#### 3.4.2. Three Months after Follow-Up

**Figure 3:**
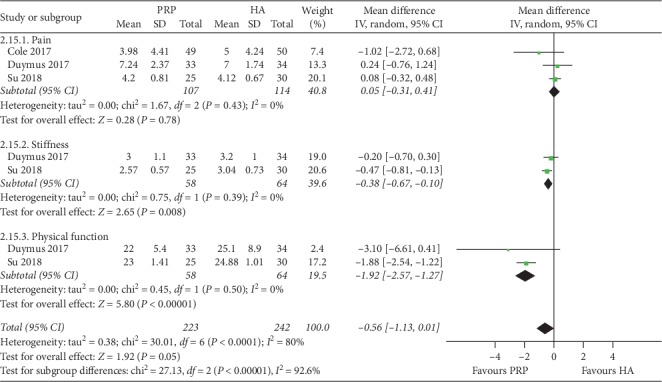
Forest plots showing the effect of PRP on WOMAC scores at 3rd months of follow-up compared with HA in overweight patients with knee OA. WOMAC: Western Ontario and McMaster Universities Osteoarthritis Index.

In the third month, a total of 3 studies [15, 16, 19] (141 patients) provided data on VAS for the PRP and HA groups, and a total of 3 studies [5, 16, 19] (221 patients) provided data on WOMAC pain score. There was no significant difference between the two groups according to the results of the pooled analysis (VAS: *P*=0.45, *I*^2^ = 72%; WOMAC pain score: *P*=0.78, *I*^2^ = 0%). A total of 2 studies (122 patients) provided data on WOMAC total score, stiffness score, and physical function score for the PRP and HA groups [16, 19]. Based on the results of the pooled analysis, there was a statistically significant difference between the two groups at WOMAC scores (total score: *P*=0.002, *I*^2^ = 0%; stiffness score: *P*=0.008, *I*^2^ = 0%; physical function score: *P* < 0.00001, *I*^2^ = 0%) (Figure 3).

#### 3.4.3. Six Months after Follow-Up

In the 6th month, a total of 3 studies [5, 16, 19] (221 patients) provided data on VAS for the PRP and HA groups, 3 studies [13, 17, 18] (433 patients) provided data on EQ-VAS, and 3 studies [5, 13, 17] (365 patients) provided data on IKDC score. There was no significant difference between the two groups according to the results of the pooled analysis of the above relevant data (VAS: *P*=0.44, *I*^2^ = 75%; EQ-VAS: *P*=0.08, *I*^2^ = 0%; IKDC: *P*=0.10, *I*^2^ = 10%).

A study with a mean BMI of ≥30 Kg/m^2^ reported the WOMAC score for the sixth month of follow-up [12]. The results of the following pooled analysis included this study. 4 studies (394 patients) reported relevant data on WOMAC total score, WOMAC stiffness score, and WOMAC physical function score during the month (total score: *P*=0.01, *I*^2^ = 88%; stiffness score: *P*=0.02, *I*^2^ = 61%; physical function score: *P*=0.06, *I*^2^ = 89%) [11, 12, 16, 19]. Based on the results of the pooled analysis of WOMAC total score and WOMAC stiffness score, there was a statistically significant difference between the two groups in overweight or obese patients with knee OA. From the overall WOMAC score and the extent of joint stiffness relief, it could be concluded that the PRP group was superior to the HA group. However, there was no significant difference between the two groups according to the result of the WOMAC physical function score in overweight or obese patients with knee OA. 5 studies (493 patients) provided data on the WOMAC pain score for the PRP and HA groups (*P*=0.01, *I*^2^ = 87%) [5, 11, 12, 16, 19]. This result indicated a statistically significant difference between the two groups, which meant that in overweight or obese patients with knee OA, the PRP group was superior to the HA group in terms of pain relief.

**Figure 4:**
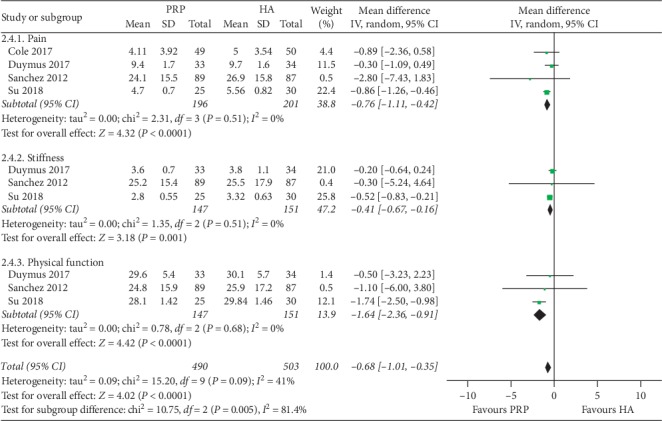
Forest plots showing the comparison of the effects of PRP and HA on WOMAC scores at 6th months of follow-up in overweight patients with knee OA.

The results of the following pooled analysis excluded this study with an average BMI of ≥30 Kg/m^2^. 3 studies (298 patients) reported relevant data on WOMAC total score, WOMAC stiffness score, and WOMAC physical function score during the month (total score: *P* < 0.00001, *I*^2^ = 23%; stiffness score: *P*=0.001, *I*^2^ = 0%; physical function score: *P* < 0.0001, *I*^2^ = 0%) [11, 16, 19]. A total of 4 studies (397 patients) provided data on the WOMAC pain score for the PRP and HA groups (*P* < 0.0001, *I*^2^ = 0%) [5, 11, 16, 19]. Based on the results of the pooled analysis of the WOMAC score, there was a statistically significant difference between the two groups in overweight patients with knee OA (Figure 4). After removing the literature with an average BMI of >30 Kg/m^2^, the heterogeneity of the WOMAC score was significantly reduced.

#### 3.4.4. Twelve Months after Follow-Up

**Figure 5:**
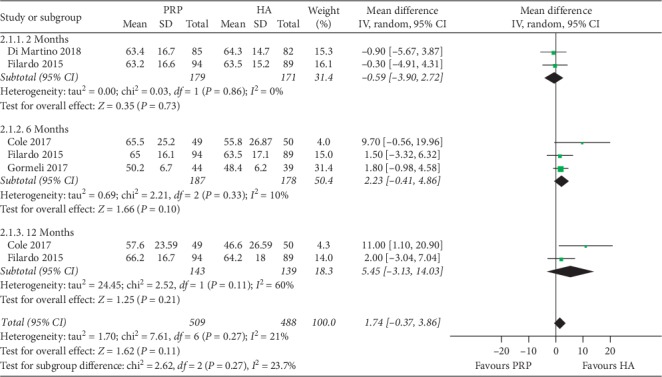
Forest plots showing the comparison of the effects of PRP and HA on IKDC score at 2nd, 6th, and 12th months of follow-up in overweight patients with knee OA. IKDC: International Knee Documentation Committee.

In the 12th month, 2 studies [13, 18] (350 patients) and 3 studies [5, 16, 19] (221 patients) provided data on EQ-VAS and VAS for the PRP and HA groups, respectively. Based on the results of the pooled analysis, there was a statistically significant difference between the two groups (EQ-VAS: *P*=0.001, *I*^2^ = 0%; VAS: *P*=0.02, *I*^2^ = 91%). This conclusion suggests that PRP is superior to HA in terms of pain relief from the 12th month of follow-up. A total of 2 studies (282 patients) provided data on the IKDC score for the PRP and HA groups [5, 13]. There was still no significant difference between the two groups according to the results of the pooled analysis (*P*=0.21, *I*^2^ = 60%). Based on the IKDC score at different follow-up months, it can be concluded that there was no statistically significant difference between the PRP group and the HA group (Figure 5).

The results of the following pooled analysis included this study with an average BMI of ≥30 Kg/m^2^. 4 studies (351 patients) reported relevant data on WOMAC total score, WOMAC stiffness score, and WOMAC physical function score during the month (total score: *P*=0.003, *I*^2^ = 94%; stiffness score: *P*=0.0007, *I*^2^ = 81%; physical function score: *P*=0.003, *I*^2^ = 94%) [12, 14, 16, 19]. Based on the results of the pooled analysis of WOMAC total score, WOMAC stiffness score, and WOMAC physical function score, there was a statistically significant difference between the two groups in overweight or obese patients with knee OA. The pooled analysis showed that the relief of joint stiffness and the recovery of physical function in the PRP group were more significant than those in the HA group. 5 studies (450 patients) provided data on the WOMAC pain score for the PRP and HA groups (*P*=0.002, *I*^2^ = 89%) [5, 12, 14, 16, 19]. This result indicated a statistically significant difference between the two groups, which meant that in overweight or obese patients with knee OA, the PRP group was superior to the HA group in terms of pain relief.

**Figure 6:**
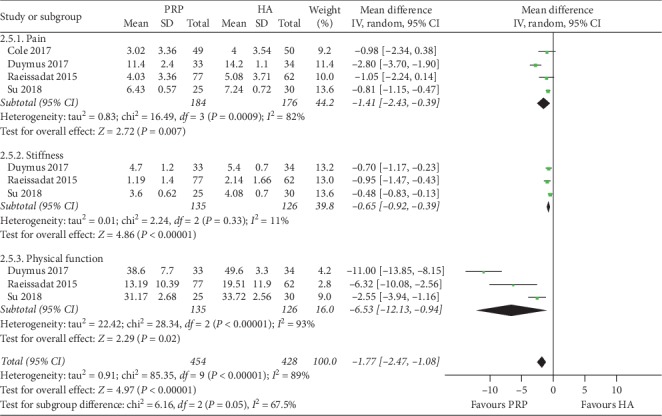
Forest plots showing the comparison of the effects of PRP and HA on WOMAC scores at 12th months of follow-up in overweight patients with knee OA.

The results of the following pooled analysis excluded this study with an average BMI of ≥30 Kg/m^2^. 3 studies (261 patients) reported relevant data on WOMAC total score, WOMAC stiffness score, and WOMAC physical function score during the month (total score:, *P*=0.02, *I*^2^ = 93%; stiffness score: *P* < 0.00001, *I*^2^ = 11%; physical function score: *P*=0.02, *I*^2^ = 93%) [14, 16, 19]. 4 studies (360 patients) provided data on WOMAC pain score for the PRP and HA groups (*P*=0.007, *I*^2^ = 82%) [5, 14, 16, 19] (Figure 6). Based on the results of the pooled analysis of the WOMAC score, there was a statistically significant difference between the two groups in overweight patients with knee OA.

### 3.5. Publication Bias

**Figure 7:**
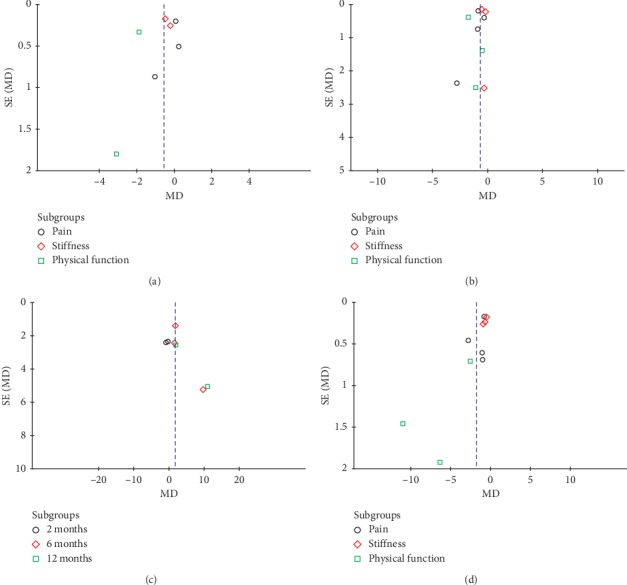
Funnel plot to detect publication bias. (a) WOMAC scores at 3rd months. (b) WOMAC scores at 6th months. (c) IKDC score. (d) WOMAC scores at 12th months.

The funnel plot is often used to assess publication bias, which is usually only performed when we have at least 10 studies. The number of studies included will have an effect on the effectiveness of the funnel plot to test publication bias. If too few studies are included, the funnel plot's testing power will decrease accordingly. As shown in Figure 7, we used funnel plots to detect publication bias (A: Related studies on WOMAC scores at 3rd months of follow-up; B: Related studies on WOMAC scores at 6th months of follow-up; C: Related studies on IKDC score; D: Related studies on WOMAC scores at 12th months of follow-up). No significant funnel asymmetry that could indicate publication bias was observed (Figures 7(a) and 7(d)). Visual inspection of the funnel plots showed asymmetry (Figures 7(b) and 7(c)). The asymmetry of the funnel plots may be due to insufficient trials and statistical heterogeneity.

### 3.6. Sensitivity Analysis

If necessary, a sensitivity analysis was conducted to identify the origins of the significant heterogeneity. Due to the high heterogeneity of the WOMAC scores, we performed a sensitivity analysis to assess the reliability of the results. When we excluded a study with an average BMI ≥30 Kg/m^2^, the heterogeneity decreased significantly. Therefore, we concluded that this study is a source of heterogeneity. At the same time, limited studies meeting the inclusion criteria may affect the reliability of the results. More high-quality RCTs are still needed in the future to compliment our conclusions.

## 4. Discussion

Obesity is associated with the prevalence and morbidity of knee OA and is considered a major risk factor [29, 30]. In obesity, being overweight can increase the joint load and have a detrimental effect on weight-bearing joints. Too much fat can cause degenerative changes in articular cartilage by subjecting it to more than biomechanical pressure [30].

HA is the most important component in synovial fluid and plays a role in the nutrition and protection of joints [31]. A large number of clinical studies have also shown that HA can alleviate joint pain and improve joint function [32]. PRP is an autogenous mixture of high concentrations of platelets and associated growth factors and other bioactive components produced by centrifugation of whole blood, which can be used to treat injuries to bones, tendons, and ligaments [33]. PRP induces chondrocyte regeneration by improving the metabolic function of the damaged structure [34], and it has been shown to have positive effects on chondrogenesis and mesenchymal stem cell proliferation [35]. Intra-articular PRP injection in patients with knee OA showed significant improvement in pain relief, symptom improvement, and quality of life [36]. This may be due to the immediate and sustained release of growth factors over a long period of time, which promotes healing and produces sustained clinical effects [37]. However, there is no consensus on the optimal ratio of PRP to various components. HA acts as lubricant and PRP provides a variety of factors to stimulate synovium and surrounding tissues. So, the combination of HA and PRP may be more effective than either method alone [38].

According to the analysis of WOMAC, EQ-VAS, VAS, IKDC, and other evaluation tools, different results can be seen in different months. Previous systematic studies have shown that PRP is an effective alternative therapy for long-term relief of knee pain and improvement of joint function [39]. However, based on the data from the literature included in the analysis, we found that the symptoms of patients in HA and PRP groups improved significantly in the first two months, and the improvement between the two groups was similar (according to WOMAC scores, EQ-VAS, and IKDC). However, after 3 months of follow-up, in WOMAC scores, the PRP group was superior to the HA group in terms of joint stiffness relief and body function recovery, but there was no difference in pain relief. At 6 months, according to VAS and EQ-VAS scores, the PRP group had no better analgesic effect than the HA group in overweight or obese patients with knee OA. In the 12th month, according to VAS, EQ-VAS and WOMAC scores, PRP was superior to HA in pain relief and functional improvement. However, there was no significant difference in the IKDC score between the PRP group and the HA group at any time. This indicated that PRP and HA had obvious alleviating effects on patients with knee OA, but the effect of reducing the severity of illness was uncertain. And PRP did not have the same effect in all stages of patients. Some researchers have found that PRP is more effective in young patients with early or moderate arthritis, but has limited effect on late OA [40]. The use of PRP can significantly improve the prognosis of patients 6 months after injection, and these improvements began in 2 months and lasted for 12 months. However, it is not clear whether the use of multiple PRP injections can lead to better outcomes [41].

In the process of analyzing the results, different results have different heterogeneity. Through careful analysis of the included studies, we find that the following reasons may be the source of heterogeneity: Firstly, the patients included in each article have different degrees of illness, and related studies have found that PRP and HA have different effects on patients with OA at different stages, such as the effect of PRP on patients with advanced knee OA is not obvious [42]. In addition, we found that the results of the study were heterogeneous in the follow-up period of 6 and 12 months, but the heterogeneity of the results was significantly reduced when the study with an average BMI >30 Kg/m^2^ was removed. This study may be the source of heterogeneity, and we classified and analyzed it. In subgroup analysis, how to choose the effect model is a problem to be solved. We find that no matter whether a fixed-effect model or a random-effect model is used in Figures 3–6, the statistical significance of the *P* value and the value of *I*^2^ have not changed, and the results are still robust. If too few pieces of literature are included, the deviation of *I*^2^ value is likely to increase, which may lead to the selection of the wrong effect model [43, 44]. In particular, when a random-effect model should be selected, a fixed-effect model is wrongly selected, and the results may deviate greatly, or even the conclusion is reversed [45, 46]. Because of the relatively limited number of studies meeting the inclusion criteria, we chose a random-effects model.

### 4.1. Limitations

Although PRP and HA have been meta-analyzed for knee OA in the past [47, 48], as far as we know, this is the first meta-analysis of the effects of HA and PRP in overweight or obese patients with knee OA, and all the studies included are RCTs, and the heterogeneity of most of the results is not high, which makes the results more accurate. Of course, this article also has its drawbacks: it only contains English systematic reviews. Non-English language literature may be neglected, leading to language bias. Because some non-RCT studies and studies of patients with an average BMI <25 Kg/m^2^ were removed during the inclusion process, the sample size of the study was not very large, which made the study have relevant deviation. Due to the limited number of studies included, the number of studies related to many outcome indicators was small during the subgroup analysis, which may result in high heterogeneity and publication bias. In addition, the dosage and frequency of drug injections used in each study were different, and multiple PRP injections were more effective for early patients than single injections [17], which also led to deviations from the results of the study. And the companies that produced PRP and HA were different, which might also be a source of heterogeneity. In addition, the methods of producing PRP and HA were different in various studies, and two independent studies had found that there might be significant biological differences between PRP preparations in single donor models [49]. In addition, different ways of injection by physicians could also have an impact on the results.

## 5. Conclusions

In overweight or obese patients with knee OA, the degree of remission between PRP and HA in the first two months was similar (WOMAC score, EQ-VAS, and IKDC). At 6 months and 12 months, PRP was better than HA in relieving pain and improving joint function. However, for the IKDC score, there was no significant difference between PRP and HA at any time, which required larger sample size to analyze and discuss.

## Figures and Tables

**Table 1:** Characteristics of all the trials included in the meta-analysis.

Authors	Country	Study type	Sample size	Mean BMI (kg/m^2^)	Mean age (years)	Gender	Radiographic classification		Intervention	Follow-up (mo)
			PRP : HA	PRP : HA	PRP : HA	M : F	K-L (I/II/III/IV)	Ahlbäck (I/II/III)		
Sánchez et al. [11]	Spain	RCTs	176 (89/87)	27.9/28.2	60.5/58.9	85/91	NP^†^	45/32/12 (PRP)^†^	3 times, 8 mL, weekly^†^	1, 2, 6
							NP^‡^	42/32/11 (HA)^‡^	3 times, weekly^‡^	

Vaquerizo et al. [12]	Spain	RCTs	96 (48/48)	30.7/31.0	62.4/64.8	38/58	2.6 (mean score)^†^	NP^†^	3 times, 8 mL, every 2 weeks^†^	6, 12
							2.8 (mean score)^‡^	NP^‡^	1 time, 60 mg/3 mL^‡^	

Raeissadat et al. [14]	Iran	RCTs	139 (77/62)	28.20/27.03	56.85/61.13	23/116	5/34/29/9^†^	NP^†^	2 times, 5 mL, monthly^†^	1, 6, 12
							0/29/23/10^‡^	NP^‡^	3 times, 20 mg/2 mL, 500 to 730 kDa, monthly^‡^	

Filardo et al. [13]	Italy	RCTs	193 (94/89)	26.6/26.9	53.32/57.55	112/71	2.0 (mean score)^†^	NP^†^	3 times, 5 mL, weekly^†^	2, 6, 12
							2.0 (mean score)^‡^	NP^‡^	3 times, 30 mg/2 mL, >1,500 kDa, weekly^‡^	

Paterson et al. [15]	Australia	RCTs	21 (11/10)	27.92/30.87	49.91/52.70	15/6	0/11 (II-III)/0^†^	NP^†^	3 times, 3 ml^†^	1, 3
							0/10 (II-III)/0^‡^	NP^‡^	3 times, 3 mL^‡^	

Duymus et al. [16]	Turkey	RCTs	67 (33/34)	27.6/28.4	60.4/60.3	2/65	0/22/11/0^†^	NP^†^	2 times, 5 mL per time, every 2 weeks^†^	1, 3, 6, 12
							0/24/10/0^‡^	NP^‡^	40 mg/2 mL, 1,600 kDa^‡^	

Görmeli et al. [17]	Turkey	RCTs	83 (44/39)	28.4/29.7	53.8/53.5	36/47	0/30 (II-III)/14^†^	NP^†^	1 time, 5 mL, weekly^†^	6
							0/25 (II-III)/14^‡^	NP^‡^	3 times, 30 mg/2 mL^‡^	

Cole et al. [5]	USA	RCTs	99 (49/50)	27.4/29.0	55.9/56.8	48/51	3/26/20/0^†^	NP^†^	3 times, 4 mL, weekly^†^	1, 3, 6, 12
							0/27/22/0^‡^	NP^‡^	3 times, 16 mg/2 mL, 6,000 kDa^‡^	

Di Martino et al. [18]	Italy	RCTs	167 (85/82)	27.2/26.8	52.7/57.5	100/67	2.0 (mean score)^†^	NP^†^	3 times, 5 mL, weekly^†^	2, 6, 12, 24
							2.0 (mean score)^‡^	NP^‡^	3 times, 30 mg/2 mL, >1,500 kDa, weekly^‡^	

Su et al. [19]	China	RCTs	55 (25/30)	28.17/28.69	54.16/53.13	23/32	0/13/12/0^†^	NP^†^	2 times, 6 ml, every 2 weeks^†^	1, 3, 6, 12, 18
							0/14/16/0^‡^	NP^‡^	2 times, 20 mg/2 mL, once a week^‡^	

^†^PRP group, ^‡^HA group. PRP: platelet-rich plasma; HA: hyaluronic acid; K-L: Kellgren and Lawrence grading scale; NP: not provided.

**Table 2:** Outcomes of the meta-analysis in different follow-up months.

Follow-up	Evaluation tools	Number of studies	Patients PRP : HA	MD	95% CI	*P* < 0.05	*I* ^2^ (%)
1 months	VAS	3	69/74	0.01	[−0.13, 0.15]	No	0
	WOMAC total score	2	58/64	−3.33	[−8.84, 2.18]	No	78
	WOMAC pain score	3	107/114	0.01	[−0.47, 0.50]	No	16
	WOMAC stiffness score	2	58/64	−0.11	[−0.47, 0.24]	No	35
	WOMAC physical function score	2	58/64	−2.35	[−5.28, 0.57]	No	59
2 months	EQ-VAS	2	179/171	2.15	[−0.57, 4.88]	No	0
	IKDC	2	179/171	−0.59	[−3.90, 2.72]	No	0
3 months	VAS	3	68/73	−0.20	[−0.71, 0.31]	No	72
	WOMAC total score	2	58/64	−1.35	[−2.19, −0.50]	Yes	0
	WOMAC pain score	3	107/114	0.05	[−0.31, 0.41]	No	0
	WOMAC stiffness score	2	58/64	−0.38	[−0.67, −0.10]	Yes	0
	WOMAC physical function score	2	58/64	−1.92	[−2.57, −1.27]	Yes	0
6 months	VAS	3	107/114	−0.35	[−1.23, 0.54]	No	75
	EQ-VAS	3	223/210	1.89	[−0.19, 3.96]	No	0
	IKDC	3	187/178	2.23	[−0.41, 4.86]	No	10
	WOMAC total score	3	147/151	−3.89	[−5.60, −2.18]	Yes	23
		4^§^	195/199^§^	−7.62^§^	[−13.51, −1.72]^§^	Yes^§^	88^§^
	WOMAC pain score	4	196/201	−0.76	[−1.11, −0.42]	Yes	0
		5^§^	244/249^§^	−1.74^§^	[−3.13, −0.36]^§^	Yes^§^	87^§^
	WOMAC stiffness score	3	147/151	−0.41	[−0.67, −0.16]	Yes	0
		4^§^	195/199^§^	−0.62^§^	[−1.12, −0.11]^§^	Yes^§^	61^§^
	WOMAC physical function score	3	147/151	−1.64	[−2.36, −0.91]	Yes	0
		4^§^	195/199^§^	−4.23^§^	[−8.58, 0.13]^§^	No^§^	89^§^
12 months	VAS	3	107/114	−1.27	[−2.36, −0.18]	Yes	91
	EQ-VAS	2	179/171	4.64	[1.86, 7.42]	Yes	0
	IKDC	2	143/139	5.45	[−3.13, 14.03]	No	60
	WOMAC total score	3	135/126	−8.79	[−16.22, −1.35]	Yes	93
		4^§^	183/168^§^	−12.11^§^	[−20.21, −4.01]^§^	Yes^§^	94^§^
	WOMAC pain score	4	184/176	−1.41	[−2.43, −0.39]	Yes	82
		5^§^	232/218^§^	−1.95^§^	[−3.18, −0.71]^§^	Yes^§^	89^§^
	WOMAC stiffness score	3	135/126	−0.65	[−0.92, −0.39]	Yes	11
		4^§^	183/168^§^	−0.99^§^	[−1.57, −0.42]^§^	Yes^§^	81^§^
	WOMAC physical function score	3	135/126	−6.53	[−12.13, −0.94]	Yes	93
		4^§^	183/168^§^	−8.90^§^	[−14.82, −2.99]^§^	Yes^§^	94^§^

^§^Include a study with BMI > 30 Kg/m^2^.
